# Interplay between global and pathway-specific synaptic plasticity in CA1 pyramidal cells

**DOI:** 10.1038/s41598-017-17161-z

**Published:** 2017-12-06

**Authors:** Sven Berberich, Jörg Pohle, Marie Pollard, Janet Barroso-Flores, Georg Köhr

**Affiliations:** 1 Central Institute of Mental Health, Medical Faculty Mannheim/Heidelberg University, J 5, 68159 Mannheim, Germany; 2 Department of Molecular Neurobiology, Max-Planck-Institute for Medical Research, Jahnstraße 29, 60120 Heidelberg, Germany; 3 Present Address: Department of Pharmacology, Heidelberg University, Im Neuenheimer Feld 366, 69120 Heidelberg, Germany; 4 Present Address: Greenville Neuromodulation Center - FHC, Inc., 179 Main Street, Greenville, PA, 16125 USA

## Abstract

Mechanisms underlying information storage have been depicted for global cell-wide and pathway-specific synaptic plasticity. Yet, little is known how these forms of plasticity interact to enhance synaptic competition and network stability. We examined synaptic interactions between apical and basal dendrites of CA1 pyramidal neurons in mouse hippocampal slices. Bursts (50 Hz) of three action potentials (AP-bursts) paired with preceding presynaptic stimulation in stratum radiatum specifically led to LTP of the paired pathway in adult mice (P75). At adolescence (P28), an increase in burst frequency (>50 Hz) was required to gain timing-dependent LTP. Surprisingly, paired radiatum and unpaired oriens pathway potentiated, unless the pre-post delay was shortened from 10 to 5 ms, which selectively potentiated paired radiatum pathway, since unpaired oriens pathway decreased back to baseline. Conversely, the exact same 5 ms pairing in stratum oriens potentiated both pathways, as did AP-bursts alone, which potentiated synaptic efficacy as well as current-evoked postsynaptic spiking. L-type voltage-gated Ca^2+^ channels were involved in mediating synaptic potentiation in oriens, whereas NMDA and adenosine receptors counteracted unpaired stratum oriens potentiation following pairing in stratum radiatum. This asymmetric plasticity uncovers important insights into alterations of synaptic efficacy and intrinsic neuronal excitability for pathways that convey hippocampal and extra-hippocampal information.

## Introduction

Hebbian, homosynaptic plasticity representing pathway-specific modifications in synapse strength has been considered an important mechanism accounting for information storage in the brain for decades^1^. Plasticity of intrinsic excitability was later recognized as a candidate memory storage mechanism^2^. By contrast, heterosynaptic plasticity, although known for a long time to accompany homosynaptic plasticity^3^, had received little attention until experimental and theoretical evidence suggested that non-Hebbian plasticity provides learning systems with stability through enhanced synaptic competition within and across dendritic compartments^4–6^.

Competition among synapses can take place in various forms. Global intracellular signalling can act as a filter for many synapses^4^ or a specific group of synapses if it is localized^7–9^. Convergent inputs can also compete for control of the timing of postsynaptic action potentials^10^. The prevailing mechanism could differ across brain regions and could also depend on the stage of development.

Global synaptic changes have been observed in a variety of neurons either contributing to homeostatic regulations secondary to homosynaptic plasticity^11^ or being induced as a primary synaptic modification in hippocampal CA1^12^, cortical layer 2/3^13^, thalamocortical relay^14^ and spinal cord lamina I^15^ neurons. Primary global synaptic changes can result from either postsynaptic depolarization^12,16,17^ or postsynaptic high-frequency APs (e.g., 100 Hz; 1 s)^12^. Synaptic plasticity can be complemented with interacting intrinsic plasticity^18^, which may even dominate depending on the induction protocol, as observed in pyramidal neurons. For example, LTP of intrinsic excitability was induced with high frequency, postsynaptic APs alone (30–40 Hz, 500 ms^19^) or in combination with prolonged, alternating presynaptic stimulation (20 Hz, 5 s^20^). It is less clear whether brief bursts of 3–5 action potentials alone, conventionally being part of spike-timing dependent plasticity protocols^21^, enhance intrinsic excitability. Brief AP-bursts alone generate no LTP on average in neocortical (cf. Fig. 8 of ref.^4^) and hippocampal CA3 neurons^22^. Still, a large scatter in the mean EPSPs across neurons may have skewed the outcome erroneously in the aforementioned studies and even constituted a condition for excluding cells^23^.

Homosynaptic, pathway-specific LTP in the hippocampus that depends on postsynaptic Ca^2+^ elevations and CaMKII activity can be induced via NMDARs or voltage-gated Ca^2+^ channels. Homosynaptic plasticity can occur together with heterosynaptic plasticity within or between apical and basal dendritic compartments of pyramidal cells^24^. In general, induction of heterosynaptic plasticity is mediated through intracellular or intercellular signaling pathways, often involving adenosine and non-neuronal cells^25–28^.

In the present study, developmental and timing aspects of pairing protocols that include brief AP-bursts were examined in CA1 pyramidal neurons from mouse hippocampal slices. In adolescent mice, repetition of brief bursts of APs alone induced a form of global LTP of excitatory postsynaptic responses. The global LTP was differently modulated by pairing AP-bursts with presynaptic stimulation in stratum radiatum (RAD) versus stratum oriens (OR), which receive distinct inputs from CA2 and CA3 areas^29–31^. Specifically, global LTP remained largely unaffected when presynaptic stimulation in OR was paired with postsynaptic AP-bursts. The exact same pairing in RAD, at 5 ms but not 10 ms pre-post delay, induced pathway-specific LTP by means of heterosynaptic plasticity across dendritic compartments in OR involving NMDA and adenosine receptor activation.

## Results

### Spike-timing dependent protocols with 10 ms pre-post delay in adult versus adolescent mice

**Figure 1:**
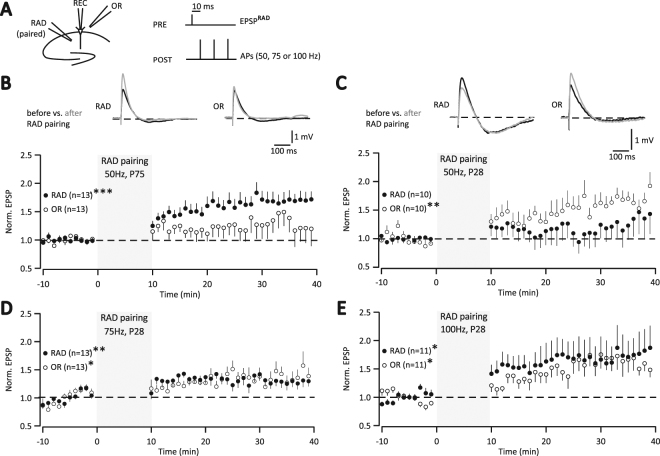
RAD pairing with 10 ms pre-post delay in adult versus adolescent mice. (**A**) Recording scheme. During induction (RAD pairing) three postsynaptic APs (“POST”) followed 10 ms after presynaptic RAD stimulation (“PRE”). (**B**) P75 mice: during induction (gray area), EPSPs were evoked 10 ms prior to 50 Hz triplet APs, repeated at 0.1 Hz for 10 min. Averages of all baseline EPSPs from example cell evoked alternately in RAD and OR before (dark line) and 20–30 min after RAD pairing (gray line). Time course of normalized averages of EPSPs ( ± SEM) (***p < 0.001). (**C**) P28 mice: RAD pairing, 50 Hz, (**p < 0.01), for further details see (B). (**D**) P28 mice: RAD pairing, 75 Hz, (***p < 0.001), for further details see (B). (**E**) P28 mice: RAD pairing, 100 Hz, (*p < 0.05), for further details see (B).

Former studies examining pathway-specific LTP in CA1 pyramidal neurons of hippocampal slices with spike-timing dependent protocols tested two pathways in stratum radiatum (RAD) (e.g.,^23^). Here, we also tested a pathway in RAD, but similar to former field recordings (e.g.,^32^) the unpaired control pathway was in stratum oriens (OR) (Fig. 1A). First, we tested triplets of APs at a frequency of 50 Hz in adult mice (P75) as previously shown for two RAD pathways^23^. The induction protocol consisted of triplet APs generated by 3 ms somatic current injections preceded (10 ms) by presynaptic stimulation, repeated 60 times at 0.1 Hz for a duration of 10 min^23^. This AP-burst pairing protocol induced pathway-specific LTP in slices of adult mice (Fig. 1B, P75: RAD, 1.67 ± 0.14, p = 0.0007; OR, 1.32 ± 0.24, p = 0.282, n = 13). Increased excitatory postsynaptic potentials (EPSPs) were observed in the unpaired pathway though these were not significant (Fig. 1B, see also Methods of^23^).

In contrast, in slices of adolescent mice (P28), we observed LTP only in the unpaired pathway with the same 50 Hz protocol as above (for adult mice) (Fig. 1C: RAD, 1.27 ± 0.28, p = 0.672; OR, 1.67 ± 0.10, p = 0.008, n = 10). Because the paired pathway did not significantly potentiate at P28 (Fig. 1C), we next increased the frequency of the AP-triplets to 75 Hz and 100 Hz (see e.g.,^33^ for 100 Hz) and exhibited LTP in both the paired and unpaired pathway (Fig. 1D, 75 Hz: RAD, 1.30 ± 0.09, p = 0.003; OR, 1.38 ± 0.14, p = 0.039, n = 13; Fig. 1E, 100 Hz: RAD, 1.74 ± 0.35, p = 0.045; OR, 1.64 ± 0.19, p = 0.011, n = 11).

At adolescence (P28), postsynaptic AP-bursts as part of paring protocols with 10 ms pre-post delay do not induce pathway-specific LTP in the paired RAD pathway. Moreover, triplet AP-burst pairing required a frequency above 50 Hz to induce LTP of the paired pathway in young mice (P28), consistent with young rats^33^. Thus, spike-timing dependent protocols established at adulthood cannot readily be applied to adolescence.

### Spike-timing dependent protocols with 5 ms pre-post delay in adolescent mice

**Figure 2:**
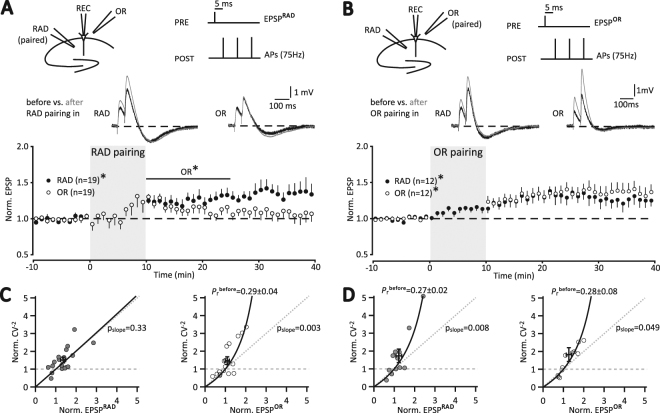
Pathway-specific LTP in apical but not in basal CA1 synapses. (**A**) Recording scheme for RAD pairing. During induction (gray area), EPSPs were evoked 5 ms prior to 75 Hz triplet APs, repeated at 0.1 Hz for 10 min. Averages of 6 EPSP examples evoked alternately in RAD and OR before (dark line) and 20–30 min after RAD pairing (gray line). Time course of normalized averages of EPSPs ( ± SEM) (*p < 0.05). Horizontal line, labeled OR^*^, indicates time interval in which amplitude change in OR was significant (*p < 0.05). (**B**) OR pairing, for further details see (A). EPSPs of the unpaired pathway were also monitored during induction. (**C**) Variance analysis of EPSP amplitude fluctuations. Normalized inverse squared coefficient of variation (CV^−2^) is plotted against normalized amplitudes of RAD (left) and OR (right). p_slope_ indicates the probability that the slope of a linear fit through the origin is unequal to 1. The expression mechanism of LTP in RAD fits best with an increase in the number of active synapses (*n*) (left), whereas the expression mechanism of LTP in OR fits best with an increase in release probability (*P*_*r*_, right, black curve, eq. 1). (**D**) CV^−2^ analyses after OR pairing indicate mainly a change in *P*_*r*_ for both pathways. Dotted and dashed gray lines illustrate the hypothesis that LTP is due to an increase in the number of active synapses *n* or in quantal size *q*, respectively.

Recent spike-timing studies often used 5 instead of 10 ms pre-post delay during pairing, e.g.^34,35^, consistent with a former study addressing input specificity of synaptic modification^36^. To examine 5 ms pre-post delay at P28, we chose an AP-burst of 75 Hz which i) is sufficient to induce LTP in RAD (Fig. 1D) and ii) is a compromise between 50 Hz used in some studies^23,37^ and 100 Hz in others^12,33^. Thus, single EPSPs were evoked 5 ms before each 75 Hz AP-triplet and repeated 60 times at 0.1 Hz. Pairing in RAD generated pathway-specific LTP (Fig. 2A: RAD, 1.37 ± 0.12, p = 0.003; OR, 1.07 ± 0.10, p = 0.480, n = 19), since EPSPs in OR increased only transiently (p = 0.003, 0.016 and 0.045 for 5, 10 and 15 min, respectively, n = 19; Fig. 2A). The time course of modulating OR after RAD pairing was not affected in distinct subsets of these 19 experiments with continuation of presynaptic stimulation in OR during RAD pairing (n = 9) or using paired-pulse stimulation before and after induction (n = 6). OR test pulses showed that this unpaired pathway increased gradually during RAD pairing (Fig. 2A). Notably, when both paired and unpaired pathways were examined in RAD^36^, input specificity was obtained without modulation of the unpaired pathway.

By strong contrast, pairing in OR exhibited LTP of EPSPs in both pathways (Fig. 2B: OR, 1.36 ± 0.10, p = 0.023; RAD, 1.26 ± 0.10, p = 0.005, n = 12) and thus failed to generate pathway-specific LTP in basal dendrites of CA1 pyramidal cells.

Next, we analyzed the fluctuations of EPSPs to estimate the expression mechanisms of LTP (see our Methods). In brief, the coefficient of variation (CV) was determined as the standard deviation of EPSPs divided by the average EPSP of the 10 min baseline period and 20 to 30 min after induction, respectively. Then the inverse squared coefficient of variation (CV^−2^) of the 20 to 30 min interval after induction was normalized to the respective baseline CV^−2^ and plotted against relative change in EPSP amplitude as in former studies (ref.^38^, their Fig. 4e and their supplements, as well as ref.^39^, their Fig. 11B). Following RAD pairing, the expression of LTP in RAD fitted best with an increase in the number of active synapses *n*, since the slope of the linear fit was not significantly different from 1 (p_slope_, Fig. 2C). By contrast, the variability of OR EPSPs after their decay to control level was mainly modulated by release probability consistent with equation (1) (Fig. 2C). Pairing in OR mainly increased the release probability *Pr* in both pathways (Fig. 2D).

Together, these pairing experiments in OR vs. RAD demonstrate asymmetric plasticity in hippocampal CA1 with pathway-specific LTP selectively in RAD, requiring modulation of the unpaired OR pathway.

### Global synaptic and intrinsic LTP induced by postsynaptic action potential bursts without presynaptic pairing

**Figure 3:**
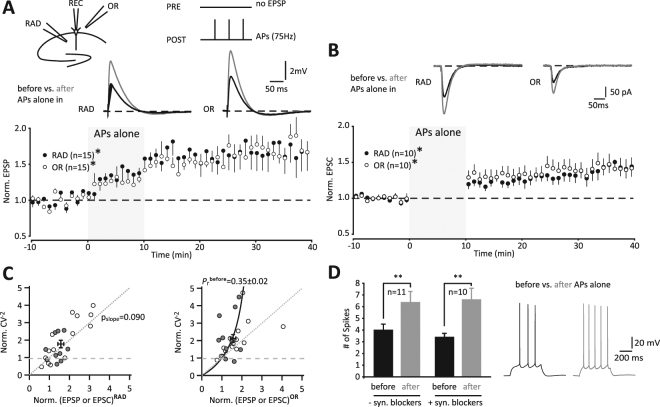
Action potential (AP) bursts alone induce global LTP of synaptic and intrinsic excitability. (**A**) Recording scheme and example cell with averages of all excitatory postsynaptic potentials (EPSPs) before and 20–30 min after induction with 75 Hz triplet APs. Time course of normalized averages of EPSPs before and after induction. EPSPs were also monitored during induction. (**B**) Example cell with averages of 6 excitatory postsynaptic currents (EPSCs) before and 20–30 min after induction. Time course of normalized averages of EPSCs before and after induction, during which 75 Hz triplet APs were generated in current-clamp with repetitions at 0.1 Hz for 10 min (RAD, filled; OR, unfilled; *p < 0.05). (**C**) Variance analysis of EPSC (gray filled) or EPSP (unfilled) amplitude fluctuations (for further details see Fig. 2C). The expression mechanism of LTP in RAD fits best with an increase in the number of active synapses (*n*) (left), whereas the expression mechanism of LTP in OR fits best with an increase in *P*_*r*_ (right, black curve, eq. 1). As a special case, the plot for OR is hardly correlated (*r* = 0.38), therefore no p_slope_ was determined. (**D**) Change of AP firing tested during 500 ms depolarization before and 30 min after induction (left columns and example traces are from a subset of A) or tested during 600 ms depolarization in the presence of glutamatergic and GABAergic receptor blockers without synaptic stimulation (right columns) (**p < 0.01, after vs. before).

After verifying in adolescent mice (P28) that presynaptic stimulations in the absence of AP triplets did not affect the amplitude of EPSPs up to 50 min (see Methods), we tested whether unpaired postsynaptic AP-bursts generate a global form of LTP, which could be modulated by paired EPSPs in RAD (Fig. 2A) but not by paired EPSPs in OR (Fig. 2B). Again, we tested AP-bursts at 75 Hz and monitored changes of EPSPs during induction and afterwards. During induction, we alternated presynaptic, electrical stimulations between RAD and OR at a 5 s delay to AP triplets to prevent their influence on EPSPs. Under these conditions, APs alone were indeed capable and sufficient to induce global LTP at apical CA1 dendrites in RAD and at basal dendrites in OR. The gradual EPSP increase during induction reached steady state after terminating induction (Fig. 3A: RAD, 1.68 ± 0.22, p = 0.0095; OR, 1.70 ± 0.21, p = 0.0051, n = 15). To validate this result, we next recorded excitatory postsynaptic currents (EPSCs) in voltage-clamp before and after generating APs alone in current-clamp. Voltage-clamp improved the stability of our baseline responses and consistently, EPSCs increased in both pathways (Fig. 3B: RAD, 1.41 ± 0.12, p = 0.009; OR, 1.41 ± 0.13, p = 0.009, n = 10). Notably, presynaptic stimulations were not required during LTP induction to obtain global LTP as tested here (Fig. 3B).

The expression of LTP in RAD fitted best with an increase in the number of active synapses, while LTP expression in OR fitted best with an increase in release probability (Fig. 3C).

Besides synaptic changes, we also observed persistent changes in excitability leading to increased spiking frequency. More precisely, as part of our EPSP recording in Fig. 3A, we injected constant current of ∼50 to 200 pA for 500 ms in order to evoke 3 to 5 APs following acquisition of baseline EPSPs. The AP firing frequency was then compared 30 min after induction using the same constant current injection (Fig. 3D left bars: before, 4.03 ± 0.48; after, 6.39 ± 0.91, p = 0.0012, n = 11). To test more directly whether this increase in AP firing is due to an increase in intrinsic excitability, we pharmacologically prevented synaptic activation of excitatory and inhibitory receptors (AMPARs, NMDARs, group I mGluRs, GABA_A_Rs and GABA_B_Rs). After induction with APs alone, AP firing frequency increased for at least 30 min (Fig. 3D right bars: before, 3.43 ± 0.29; after, 6.63 ± 0.94, p = 0.008, n = 10). This increase in AP firing was not observed in control experiments in the absence of AP-bursts (baseline, 3.9 ± 0.3 APs; after, 3.9 ± 0.5 APs; p = 0.90, n = 8).

In summary, AP-bursts globally and persistently potentiated spiking frequency of CA1 pyramidal neurons as well as synaptic efficacy, the latter via increasing the release probability in OR and the number of active synapses in RAD. Of note, pairing in OR or RAD (Fig. 2) did not change these mechanisms in the respective paired pathways, when compared with APs alone.

### NMDA receptor-dependent induction of pathway-specific plasticity

**Figure 4:**
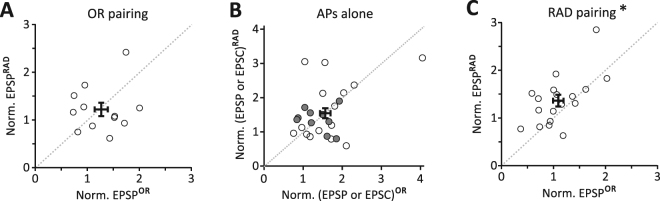
Pathway specificity. (**A**) Summary regarding pathway specificity for neurons illustrated in Figs 2 and 3 for OR pairing, (**B**) APs alone including EPSCs (gray filled) and EPSPs (unfilled) and (**C**) RAD pairing (*p = 0.02). Plots are normalized RAD EPSPs against OR EPSPs of the last 10 min of recording per cell.

Following OR pairing and AP-burst induction alone, there is no obvious difference in the course of LTP development. Pairwise comparisons of normalized RAD EPSPs with normalized OR EPSPs were similar for OR pairing (p = 0.677, n = 12, Wilcoxon signed-rank test (WSRT); Fig. 4A) or APs alone (p = 0.979, n = 25, WSRT; Fig. 4B). Following RAD pairing, LTP was pathway-specific with respect to pairwise comparisons between RAD and OR normalized EPSPs (p = 0.015, n = 19, WSRT; Fig. 4C).

**Figure 5:**
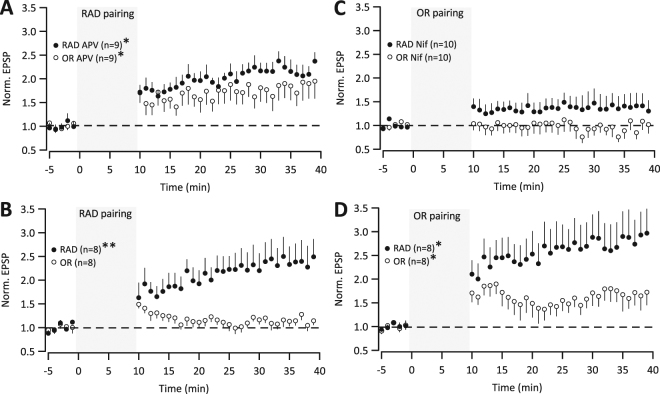
NMDAR and L-type Ca^2+^ channel antagonism. (**A**) RAD pairing in D-APV (50 µM). During induction (gray), EPSPs were evoked in RAD 5 ms prior to 75 Hz triplet APs, repeated at 0.1 Hz for 10 min (*p < 0.05). (**B**) RAD pairing in absence of D-APV. (**C**) OR pairing in Nifedipine (10 µM). During induction (gray), EPSPs were evoked in OR 5 ms prior to 75 Hz triplet APs, repeated at 0.1 Hz for 10 min. (**D**) OR pairing in absence of Nifedipine.

Given that NMDARs are frequently involved in the induction of input-/pathway-specific synaptic plasticity^1^, we tested RAD pairing when NMDARs were antagonized. In the presence of D-APV (50 µM), RAD EPSPs and OR EPSPs potentiated, eliciting global LTP (Fig. 5A: RAD, 2.19 ± 0.17, p = 0.0013; OR, 1.83 ± 0.30, p = 0.033, n = 9). This NMDAR-independent global LTP is reminiscent of NMDAR-independent LTP in the visual cortex^40^. The higher potentiations compared with Figs 1–4 are consistent with higher pipette series resistances in this set of pharmacological experiments (see Methods). Nevertheless, we performed matching control experiment in the absence of D-APV, finding the unpaired OR pathway modulated as in Fig. 2A (Fig. 5B: RAD, 2.39 ± 0.37, p = 0.007; OR, 1.14 ± 0.10, p = 0.17, n = 8).

To examine whether induction of global LTP involved L-type voltage-gated Ca^2+^ channels activated by backpropagating APs, we tested OR pairing in the presence of nifedipine (10 µM). Neither RAD EPSPs nor OR EPSPs were enhanced following OR pairing (Fig. 5C: RAD, 1.39 ± 0.26, p = 0.16; OR, 0.96 ± 0.15, p = 0.24, n = 10), consistent with NMDAR-independent mechanisms^41,42^. In the absence of nifedipine, RAD EPSPs and OR EPSPs were enhanced (Fig. 5D: RAD, 2.81 ± 0.52, p = 0.01; OR, 1.70 ± 0.23, p = 0.02; n = 8; see also Fig. 2B).

### Adenosine receptors

Adenosine enzymatically derived from astrocytic ATP^43^ or pyramidal neurons^44,45^ is known to regulate the dynamic range for LTP generation, involving the high-affinity A_1_ and A_2A_ adenosine receptors (A_1_Rs and A_2A_Rs)^25,27,44,46^, with A_1_Rs having about a twofold higher affinity for adenosine than A_2A_Rs^47^. Lower adenosine concentrations decrease glutamate release by predominantly activating A_1_Rs tonically, while higher adenosine concentrations increase glutamate release via facilitatory A_2A_Rs^46,48^. Hence, we investigated whether the dualistic nature of these two adenosine receptor subtypes impinged upon the observed plasticity in OR generated by RAD pairing.

**Figure 6:**
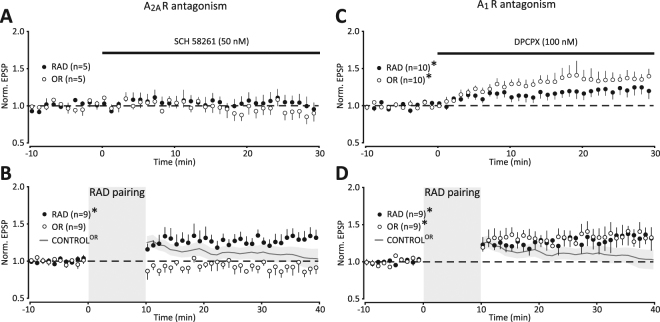
Adenosine A_2A_ receptor antagonist abolishes any potentiation in OR, and adenosine A_1_ receptor antagonist turns transient into persistent potentiation in OR. (**A**) Time course of normalized EPSPs evoked at either RAD (filled) or OR (unfilled) at 0.1 Hz before and after continuous perfusion of the A_2A_R antagonist SCH-58261 (50 nM; 30 min). (**B**) Time course of normalized averages of EPSPs before and after RAD pairing in the presence of 50 nM SCH-58261. For comparison the continuous gray line ± shaded SEM shows normalized OR EPSPs from Fig. 2A (*p < 0.05). (**C**) Time course of normalized EPSPs evoked at either RAD (filled) or OR (unfilled) at 0.1 Hz before and after continuous perfusion of the A_1_R antagonist DPCPX (100 nM; 30 min). (**D**) Time course of normalized averages of EPSPs before and after RAD pairing in the presence of 100 nM DPCPX. For comparison, the continuous gray line ± shaded SEM of normalized OR EPSP from Fig. 2A (*p < 0.05).

Consistent with a previous study^49^, the A_2A_R-specific antagonist SCH-58261 (50 nM) did not change basal synaptic transmission (Fig. 6A: RAD, 1.05 ± 0.10, p = 0.79; OR, 0.94 ± 0.09, p = 0.87, n = 5). RAD pairing in the presence of SCH-58261 led to LTP of RAD EPSPs (Fig. 6B: RAD, 1.28 ± 0.10, p = 0.008, n = 9), but not OR EPSPs (Fig. 6B: OR, 0.92 ± 0.07, p = 0.23, n = 9). Even immediately after the induction period, there was no increase in OR EPSP. Thus, the pronounced pathway-specific LTP suggests that the transient OR EPSP increase apparent in the absence of SCH-58261 (Figs 2A and 5B) was A_2A_R-mediated. In the presence of the A_2A_R-specific antagonist, A_1_R-mediated tonic inhibition could be emphasized^48^. Consistent with a tonic inhibitory effect, perfusion of the A_1_R antagonist DPCPX (100 nM) increased basal synaptic transmission (Fig. 6C: RAD, 1.21 ± 0.08, p = 0.028; OR, 1.38 ± 0.09, p = 0.010, n = 10). Next and in the presence of DPCPX, RAD pairing resulted in global LTP (Fig. 6D: RAD, 1.33 ± 0.14, p = 0.036; OR, 1.34 ± 0.13, p = 0.009; n = 9). Thus, A_1_Rs are involved in counteracting LTP in OR following RAD pairing.

### GABA_B_ receptors

GABA_A_Rs were blocked in our experiments and could not contribute to the transient increase in OR EPSPs during RAD pairing. On the other hand, GABA_B_R activation in astrocytes has been shown to mediate synaptic depression of nontetanized hippocampal synapses within apical dendrites through adenosine^28^.

Perfusion of the GABA_B_R antagonist CGP 55845 (2 µM) under baseline condition suggested that GABA_B_R activation may be more prominent in RAD than in OR, since RAD EPSPs but not OR EPSPs increased in the presence of CGP 55845 (RAD, 1.31 ± 0.07, p = 0.005; OR, 1.05 ± 0.10, p = 0.696; n = 6; Supplementary Figure S1A). Next, we examined the effects of CGP 55845 on synaptic responses to AP-bursts alone to test whether GABA_B_Rs were involved in controlling adenosine release that can occur through excitatory autoregulation^44^. In the presence of CGP 55845, OR EPSPs as well as RAD EPSPs remained potentiated throughout 30 min (RAD, 1.34 ± 0.08, p = 0.020; OR, 1.47 ± 0.10, p = 0.0034; n = 7; Supplementary Figure S1B). RAD pairing in the presence of CGP 55845 (Supplementary Figure S1C) still potentiated RAD EPSPs throughout 30 min (1.36 ± 0.09, p = 0.010, n = 11) and OR EPSPs for 4 min (1.23 ± 0.08, p = 0.049, n = 11) but not subsequently (p = 0.055, 0.089, 0.075 and 0.14 (n = 11) for the first 5, 10, 15 and 20–30 min after induction, respectively, n = 11). Thus, LTP of RAD EPSPs and transient plasticity of OR EPSPs was retained in the presence of CGP 55845. This was substantiated by the lack of change in CV^−2^ analyses in the presence (Supplementary Figure S1D) and absence of CGP 55845 (Fig. 2C). These results suggest that adenosine independent of GABA_B_R activation mainly modulated the plasticity in OR.

## Discussion

Our findings identify a pathway-specific modulation of global plasticity in apical but not basal dendrites of CA1 pyramidal cells. Global LTP was generated exclusively by postsynaptic burst activity. When brief AP-bursts were paired with prior subthreshold stimulation in stratum oriens (OR), global LTP remained largely unaffected, whereas prior subthreshold stimulation in stratum radiatum (RAD) resulted in pathway-specific LTP (with 5 ms but not with 10 ms pre-post delay).

Lack of pathway-specific LTP following OR pairing indicated that postsynaptic burst activity alone remained decisive in inducing global synaptic LTP. Alike, positive as well as negative time delays of burst pairing protocols induced LTP at apical CA1 dendrites^50^. The similarity of global LTP induced via OR pairing and via burst activity alone was further supported by the sensitivity of postsynaptic responses of both pathways to a blocker of L-type voltage-gated Ca^2+^ channels, consistent with previous studies. For example, postsynaptic theta-burst spiking alone (5 APs at 100 Hz repeated 10 times at 5 Hz) substantially and simultaneously increased synaptic currents evoked in two independent pathways in apical CA1 dendrites^33^. Similarly, repeated postsynaptic depolarizations or 1 s AP trains at 100 Hz induced global LTP of spontaneous synaptic currents^12^. The latter study suggested pre- and postsynaptic mechanisms in the generation of global LTP evidenced by an effect on CaMKII inhibition; decreased paired-pulse ratios and increased frequency and amplitude of miniature synaptic currents. Our CV^−2^ analyses following burst activity alone indicated an increase in the number of active synapses in stratum radiatum as well as an increase in release probability in stratum oriens, but no hint for conventional insertion of AMPA receptors into active postsynaptic sites. Thus, LTP is not expressed by an increase in quantal size *q*, if somatic spikes are generated either by somatic current injection as in our study and others^12,34^ or by theta burst stimulation of synaptic inputs^51^. Remarkably, after OR pairing global LTP was preferentially expressed via increased release probability.

In our study, RAD pairing led to a pathway-specific LTP if postnatal development was within the adolescent age (P28) and AP-bursts were immediately (5 ms) preceded by presynaptic stimulation in stratum radiatum. In Xenopus retinotectal connections it is known that LTP pathway specificity emerges with development^52^, which is also evident from studies in rodents and many other species. Buchanan and Mellor^33^ failed to induce pathway-specific LTP in juvenile (P14) rat slices though a later developmental stage (P45-P55) resulted in a stronger increase in the test than in the control pathway (their Figs 1C and 2C). Increases in control pathways have been observed previously when postsynaptic AP-burst activity was part of pairing protocols in rat and mouse slices (P42-P70)^8,33,53^. Still, the test pathways paired with theta-burst postsynaptic activity increased to a greater extent than the unpaired control pathways, reflecting pathway specificity. Increases in control pathways are probably underestimated, since control pathways are often not illustrated under all experimental conditions examined^50,54^ (see however control pathways in^55^) or changes in the control pathway lead to exclusion^23^. Thus, postsynaptic burst activity can affect synaptic efficacy in the absence of glutamatergic and GABAergic presynaptic activity, which we confirmed here with brief AP-bursts being part of pairing protocols. By contrast, postsynaptic single spikes are less influential in inducing global plasticity as shown in juvenile slices ( < P14) in which pathway-specific LTP was induced^54^. Interestingly, pairing protocols with 5 ms pre-post delay (and^35^ our Figs 2 and 5) allowed the generation of pathway specificity at P28 but exclusively with presynaptic stimulation in RAD (not OR, our Figs 2 and 5).

Pathway-specific LTP following RAD pairing is generally comparable with NMDAR-dependent LTP that is often studied for two CA1 inputs within apical dendrites^1^. Therefore, one wonders why NMDARs in basal dendrites failed to generate pathway-specific LTP in our OR pairing experiments. This was initially very surprising, since pathway-specific LTP can be induced in basal dendrites of CA1 pyramidal neurons as known from extracellular field recordings^24,56,57^ and from whole-cell recordings^9^. In the latter study, pathway-specific LTP was assured by local synaptic depolarization and/or dendritic spikes evoked with synaptic stimulation rather than somatic current injection^9,58^. In extracellular field recordings, synaptic stimulation likely generated backpropagating APs with reduced incidence and variable timing precision, since APs generated by repeated high-frequency electrical stimulation in either apical or basal dendrites generated plasticity across compartments^24^. Effects across compartments were also observed in our pairing experiments. Pairing in RAD, but not pairing in OR, generated pathway-specific LTP and thus, our pairing protocols generated asymmetric interactions between the two pathways. Such asymmetric modulation of plasticity has been observed previously in area CA1. High frequency priming stimulations in OR inhibited subsequent LTP in RAD but not vice versa^26^. The latter LTP-weakening effect in RAD involved muscarinic M1 acetylcholine receptor (M1R) activation. Unlike priming, release of acetylcholine following repetitive electrical stimulation in OR, either induced with high frequency stimulation^59^ or with a spike-timing dependent protocol^60^, has been shown to enhance LTP in RAD. Consistently, theta burst stimulation in RAD activated M1Rs and potentiated CA1 synaptic transmission that occluded LTP, based on recent experiments with selective M1R agonists and M1R knockout mice^61^. Thus, electrical stimulation of cholinergic fibers unlikely contributed to generate pathway-specific LTP during RAD pairing. This view is supported by the fact that OR EPSPs were either not evoked or temporally separated by a 5 s interval from the AP-bursts during induction in RAD. In contrast, cholinergic modulation required a substantially shorter interval (10 ms) to generate transient depression in CA1^62^.

We pharmacologically characterized pathway-specific LTP following RAD pairing. Antagonism of NMDARs or antagonism of A_1_Rs prevented pathway-specificity and resulted in global LTP in hippocampal CA1 (see^40^ for NMDAR-independent global LTP in the visual cortex), since the potentiation in OR synapses following RAD pairing persisted throughout the recording. Similarly, A_1_Rs were reported to destabilize LTP at OR synapses to a greater extent than LTP at RAD synapses^63^. Thus, Schaffer collateral stimulation in RAD may mediate the heterosynaptic plasticity in OR, i.e. across compartments in the basal dendrites via NMDAR-dependent A_1_R activation. Interestingly, NMDARs and A_1_Rs also mediated transient heterosynaptic depression within the RAD pathway^27,28^, whereas A_1_R-mediated heterosynaptic depotentiation in RAD following perforant path stimulation did not depend on NMDARs^64^. Thus, distinct heterosynaptic mechanisms appear to exist within apical dendritic compartments for cortical pathways in stratum lacunosum moleculare versus hippocampal pathways in RAD. The respective heterosynaptic mechanisms including its time dependence (5 ms vs. 10 ms pre-post delay) remain unknown with respect to cortical, hippocampal and septal pathways that converge within the basal dendritic compartment.

The main source of adenosine mediating the heterosynaptic plasticity at OR synapses after RAD pairing is not consistent with previously described NMDAR activation in interneurons and subsequent GABA_B_R activation in astrocytes^28^, since heterosynaptic plasticity in OR was not prevented by the GABA_B_R antagonist in contrast to the NMDAR antagonist. Though the transient potentiation in OR was shortened in the presence of a GABA_B_R antagonist, suggesting a reduced adenosine release and thus reduced A_2A_Rs contribution. This points to NMDAR-dependent adenosine release independent of GABA_B_R activation, e.g. via a direct activation of ionotropic or metabotropic glutamate receptors in astrocytes and/or neurons^65^. As expression of functional NMDARs in hippocampal astrocytes is not confirmed^66^, NMDARs rather mediate adenosine release from neurons. Indeed, neuronal adenosine released by excitatory neurons in this case has been shown to inhibit excitatory inputs through A_1_Rs via an autonomic feedback mechanism within one second^44^. Short-term depression via this auto-A_1_R^44^ might lead to LTD, if any long-term plasticity evolves. Under our conditions and following RAD pairing, however, EPSPs increased in OR through A_2A_Rs most likely by attenuating the tonic inhibitory effect of A_1_Rs as observed by others^46,48^. The subsequent decay of OR EPSPs to control level within minutes likely reflects restoration of tonic inhibition once A_2A_Rs desensitize^67^. By contrast, A_2A_R desensitization could be weaker during OR pairing than during RAD pairing, since electrical stimulation in stratum oriens elevates extracellular adenosine less than electrical stimulation in stratum radiatum^68^. Interestingly, adenosine release during OR stimulation involves L-type voltage-gated Ca^2+^ channels and/or Ca^2+^-induced Ca^2+^ release^68^, and could explain why a blocker of L-type voltage-gated Ca^2+^ channels reduced OR pairing induced LTP. Thus, distinct pathways appear capable to elevate extracellular adenosine in CA1 (NMDA in RAD and ‘Ca^2+^’ in OR) and could be involved in the timing-dependent, asymmetric plasticity in CA1.

Hebbian synaptic plasticity is associative and usually pathway-specific, and is therefore assumed to support learning and memory storage better than non-associative global plasticity. The latter can represent neuron-wide changes in synaptic efficacy and intrinsic excitability as confirmed here for CA1 pyramidal cells. Global plasticity was not observed for CA3 pyramidal neurons^22^, which express plasticity differently from CA1 pyramidal neurons^69^. However, neuronal network models often consider interactions of pathway-specific and global plasticity^6,13^. These interactions are considered to increase the repertoire of plasticity mechanisms and thereby the possibilities of learning and memory storage mechanisms. Our finding that synaptic activity in distinct CA1 pathways is capable of asymmetrically regulating global plasticity highlights that individual synapses are not regulated in isolation. The interplay between OR and RAD reflects the interaction of contextual and spatial representations important for episodic memory^70^.

## Methods

Experimental procedures were in accordance with the animal welfare guidelines of the Max Planck Society and the “European Union’s Directive 86/609/EEC”. All experimental protocols were approved by the Regional Board Karlsruhe (35-9185.81/G-273/12).

### Animals, brain slices and solutions

Acute transverse slices (~250–280 µm) from the middle hippocampus were prepared from isoflurane-anesthetized P27-P30 and P74-P77 C57Bl/6 N mice (Charles River). Brains were ice-cold perfused, cut (HM 650 V microtome, Microm International, Walldorf, Germany) and stored (~35 °C for ~30 min, then room temperature) in a chamber with sucrose saline (in mM: 87 NaCl, 25 NaHCO_3_, 2.5 KCl, 1.25 NaH_2_PO_4_, 7 MgSO_4_, 0.5 CaCl_2_, 10 glucose, 65 sucrose, 0.01 sodium pyruvate bubbled with 95% O_2_ / 5% CO_2_, ~300 mOsm). Alternatively, brains were placed and cut in ice-cold modified artificial cerebrospinal fluid (ACSF) (in mM): 125 NaCl, 25 NaHCO_3_, 2.5 KCl, 1.25 NaH_2_PO_4_, 6 MgCl_2_, 1 CaCl_2_, 3 myo-inositol, 2 sodium pyruvate, 0.4 ascorbic acid and 25 glucose bubbled with carbogen (95% O_2_, 5% CO_2_), maintained in standard ACSF (in mM: 125 NaCl, 25 NaHCO_3_, 2.5 KCl, 1.25 NaH_2_PO_4_, 1 MgCl_2_, 2 CaCl_2_, 25 glucose) at ~35 °C for ~30 min and subsequently stored at room temperature for at least 30 min prior to the start of recording. Internal solution consisted of (in mM): ~130 K-gluconate, 10 HEPES, 10 Na_2_-phosphocreatine, 0 or 10 Na-gluconate, 4 Mg-ATP, 0.3 Na-guanosine-triphosphate, 0 or 4 NaCl, 0.2 EGTA adjusted to pH 7.2 with KOH and ~280 or ~300 mOsm with K-gluconate. No difference was observed between patch pipette solutions containing 20.3 mM or 34.3 mM Na^+^.

### Recording conditions

Slices were imaged using an upright Zeiss Axioskop 2 (Göttingen, Germany) combined with a CCD camera 2400 (Hamamatsu, Herrsching, Germany). Recordings were performed at 31°−33 °C in a submerged chamber perfused at 1–2 ml/min with oxygenated standard ACSF (see above) of 300–320 mOsm (12 or 25 mM glucose) containing the NMDAR co-agonist glycine (10 µM) and the GABA_A_ antagonist gabazine (SR-95531; Sigma-Aldrich, Germany) at 5 µM, at which we did not observe epileptiform activity. Whole-cell patch-clamp recordings were performed either with an Axoclamp 2B amplifier (Molecular Devices, Berkshire, UK), an EPC9 or an EPC10 amplifier (HEKA, Lambrecht, Germany), interfaced to HEKA Patchmaster software. Data were sampled at 10 or 20 kHz and low-pass filtered at 3 kHz or 7.5 kHz. Recording pipettes (4–6 MΩ) were pulled from 0.15 mm (exception Fig. 5: 0.5 mm) walled borosilicate glass with an outside-diameter of 1.5 mm (exception Fig. 5: 2.0 mm) (Hilgenberg, Malsfeld, Germany) on a Flaming/Brown puller P-97 (Sutter Instruments, Novato, CA). Higher series resistances with the thicker glass likely restricted washout of cell dialysate thereby supporting the extent of potentiations observed in Fig. 5.

CA1 pyramidal cells were identified by firing pattern and had a resting membrane potential (*V*_rmp_) of −66.6 ± 0.3 mV and input resistance (*R*_in_) of 146.0 ± 4.2 MΩ (n = 185) both measured in current-clamp during baseline. Cells were excluded from analysis if *V*_rmp_ was more positive than −60 mV at the beginning of the recording, if *V*_rmp_ changed > 5 mV or if *R*_in_ changed > 20% during the recording. Overall, *R*_in_ changes which were monitored with hyperpolarizing pulses (−3 pA or −10 pA; 200–500 ms) leveled out.

For electrical stimulation, borosilicate glass pipettes (<3 µm; 0.5 mm walled, 2 mm outside diameter) filled with ACSF were placed in RAD, ~150 µm away from the pyramidal layer shifted in the direction of CA3 and in OR, ~50 µm away from the pyramidal layer shifted in the direction of subiculum. Pathway independence was confirmed in some experiments using cross-facilitation with 50 ms interstimulus intervals^24^. Stimulation intensity was adjusted to evoke excitatory postsynaptic potentials and currents (EPSPs and EPSCs) with similar amplitudes (RAD EPSP, 3.09 ± 0.07 mV,; OR EPSP, 2.99 ± 0.10 mV, n = 185; p = 0.28 paired t-test; RAD EPSC, −139.1 ± 26.9 pA; OR EPSC, −102.9 ± 12.3 pA, p = 0.13 paired t-test, n = 10).

Baseline EPSPs were recorded at 0.1 Hz by alternating stimulation between the two pathways (interstimulus interval, 5 s; or 0.3 s in Fig. 5). In some experiments, we stimulated paired-pulses with 50 ms interval (Figs 2, 6 and Supplementary Figure 1). Paired-pulse ratios were initially analyzed to determine pre- or postsynaptic effects of GABA_B_ or adenosine receptor antagonists and to address expression mechanisms of LTP. For the latter, we focused on the coefficient of variation (CV^−2^) of the first EPSP amplitudes (see Data Analysis) and omitted results based on paired-pulse ratios. Following baseline recording (10 min; 5 min in Fig. 5), induction protocols were initiated within 20 min after establishing whole-cell configuration. The action potential (AP) alone induction protocol consisted of triplet APs at 75 Hz induced by 3 ms somatic current injections (~1.0–1.5 nA) with 60 repetitions at 0.1 Hz for 10 min. The pairing protocol consisted of an EPSP evoked at one of the two pathways, i.e. either in stratum radiatum (RAD pairing) or in stratum oriens (OR pairing) 5 ms prior to the triplet APs and was also repeated 60 times at 0.1 Hz for 10 min (10 ms pre-post time in Fig. 1). Following induction, recordings of EPSPs at the two pathways were resumed at 0.1 Hz for 30 min. Without induction, EPSPs remained constant if evoked at 0.1 Hz for 50 min (RAD EPSP, 1.03 ± 0.07; p = 0.65, n = 6; OR EPSP, 1.05 ± 0.04; p = 0.32, n = 6; not illustrated). In one set of experiments (Fig. 3B), we recorded under the same conditions excitatory postsynaptic currents (EPSCs) in voltage-clamp (holding potential −70 mV, liquid junction potential was not corrected) during baseline and following induction, and then switched to current-clamp only during induction.

### Data Analysis

All experiments were analyzed in Fitmaster (HEKA, Lambrecht, Germany), IGOR Pro version 5 and 6 (Wavemetrics, Lake Oswego, OR, USA) and Microsoft Excel. EPSP peak amplitudes were normalized to the average of the 10 min baseline period (norm. EPSP, mean ± SEM). Statistical analysis was performed in GraphPad Prism Version 5.02 (GraphPad Software, Inc., San Diego, CA, USA). Statistical significance for LTP of EPSP/Cs was tested for the last 10 min of recording (‘after’) relative to baseline (‘before’), using two-tailed paired t-test on the absolute values. Differences in AP firing before and after pairing were tested by a two-tailed paired t-test.

To determine the expression mechanisms of LTP, normalized inverse square of the coefficient of variation (CV^−2^) of EPSP amplitudes during baseline (‘before’) and 20–30 min after plasticity induction (‘after’) was plotted against normalized EPSP amplitude (cf. Fig. 11B in ref.^39^). If paired pulses were stimulated, the first EPSP was used. Except for Fig. 3C (right panel), CV^−2^ analyses (Figs 2, 3 and Supplementary Figure 1) contain linearly correlated data. The averages are either above or on the line through the origin. Thus, without hints for changes in quantal size, we considered changes in the synaptic release probability *P*_r_ or in the number of active synapses *n*. To consider LTP through an increase in *n* (i.e. EPSP_norm_ = *n* ^after^/*n* ^before^), we used t statistics of linear regression statistics (Igor Pro 6.37) to test if the slope of a linear fit through the origin was significantly different from 1, with the p value p_slope_ given in the figures. If p_slope_ < 0.05, we tested for pure changes in *P*_r_, (i.e. EPSP_norm_ = *P*_r_^after^/*P*_r_^before^). In this case, we can fit normalized CV^−2^:

1 $$\begin{array}{ccc}{{\rm{CV}}}_{{\rm{norm}}}^{-2} & = & \frac{{{\rm{CV}}}_{{\rm{after}}}^{-2}}{{{\rm{CV}}}_{{\rm{before}}}^{-2}}=\frac{{n}_{{\rm{after}}}\times {P}_{{\rm{r}}}^{{\rm{after}}}}{1-{P}_{{\rm{r}}}^{{\rm{after}}}}\times \frac{1-{P}_{{\rm{r}}}^{{\rm{before}}}}{{n}_{{\rm{before}}}\times {P}_{{\rm{r}}}^{{\rm{before}}}}\\  & = & \frac{{P}_{{\rm{r}}}^{{\rm{after}}}}{{P}_{{\rm{r}}}^{{\rm{before}}}}\times \frac{1-{P}_{{\rm{r}}}^{{\rm{before}}}}{1-{P}_{{\rm{r}}}^{{\rm{before}}}\times {P}_{{\rm{r}}}^{{\rm{after}}}\div{P}_{{\rm{r}}}^{{\rm{before}}}}\\  & = & {{\rm{EPSP}}}_{{\rm{norm}}}\times (\frac{1-{P}_{{\rm{r}}}^{{\rm{before}}}}{1-{P}_{{\rm{r}}}^{{\rm{before}}}\times {{\rm{EPSP}}}_{{\rm{norm}}}})\end{array}$$

As usual, CV^−2^ = n*P_r_/(1 − *P*_r_) (cf. Fig. 4e and Supplementary Methods of ref.^38^). In the figures, *P*_r_ fits are only illustrated if convergent and if the fit parameter *P*_r_^before^ is ~30% as in^71^.

### Drugs

Where applicable, CGP 55845 (2 µM; Sigma-Aldrich, Germany), DPCPX (100 nM; Biotrend) or SCH 58261 (50 nM, Tocris Cookson, Bristol, UK) was bath perfused to selectively block GABA_B_, A_1_ or A_2A_ receptors, respectively. Bath perfusion of NBQX (10 μM; Biotrend, Germany), D-APV (50 µM; Tocris Cookson, Bristol, UK) or S-MCPG (500 μM; Biotrend) was used to block AMPARs, NMDARs or metabotropic GluRs, respectively. Nifedipine (10 µM; Sigma-Aldrich, Germany) was used to block L-type voltage-gated Ca^2+^ channels.

## Electronic supplementary material

Supplementary Information
