# Supplementary material for: Interplay between global and pathway-specific synaptic plasticity in CA1 pyramidal cells
Source: Sci Rep. 2017 Dec 6;7:17040. doi: 10.1038/s41598-017-17161-z (PMC5719010; doi:10.1038/s41598-017-17161-z)
Supplement: Supplementary file 1 — Supplementary Information [file 41598_2017_17161_MOESM1_ESM.pdf]

Supplementary Information for:

Interplay between global and input-specific synaptic plasticity in CA1 pyramidal cells

Sven Berberich[1,2*], Jörg Pohle[1,3*], Marie Pollard[2,4*], Janet Barroso-Flores[1] & Georg Köhr[1,2]

[1]Central Institute of Mental Health, Medical Faculty Mannheim/Heidelberg University, J 5, 68159 Mannheim, Germany

Keywords:

pathway interactions, expression mechanism of LTP, intrinsic excitability, NMDA receptors, adenosine receptors

[*]equal contribution; [2]Where the project started: Department of Molecular Neurobiology, Max-Planck-Institute for Medical Research, Jahnstraße 29, 60120 Heidelberg, Germany; [3]Present address: Department of Pharmacology, Heidelberg University, Im Neuenheimer Feld 366, 69120 Heidelberg, Germany. [4]Present address: Greenville Neuromodulation Center – FHC, Inc., 179 Main Street, Greenville, PA, 16125, USA. Correspondence and requests for materials should be addressed to G.K. (georg.koehr@zi-mannheim.de)

Supplementary Figure S1

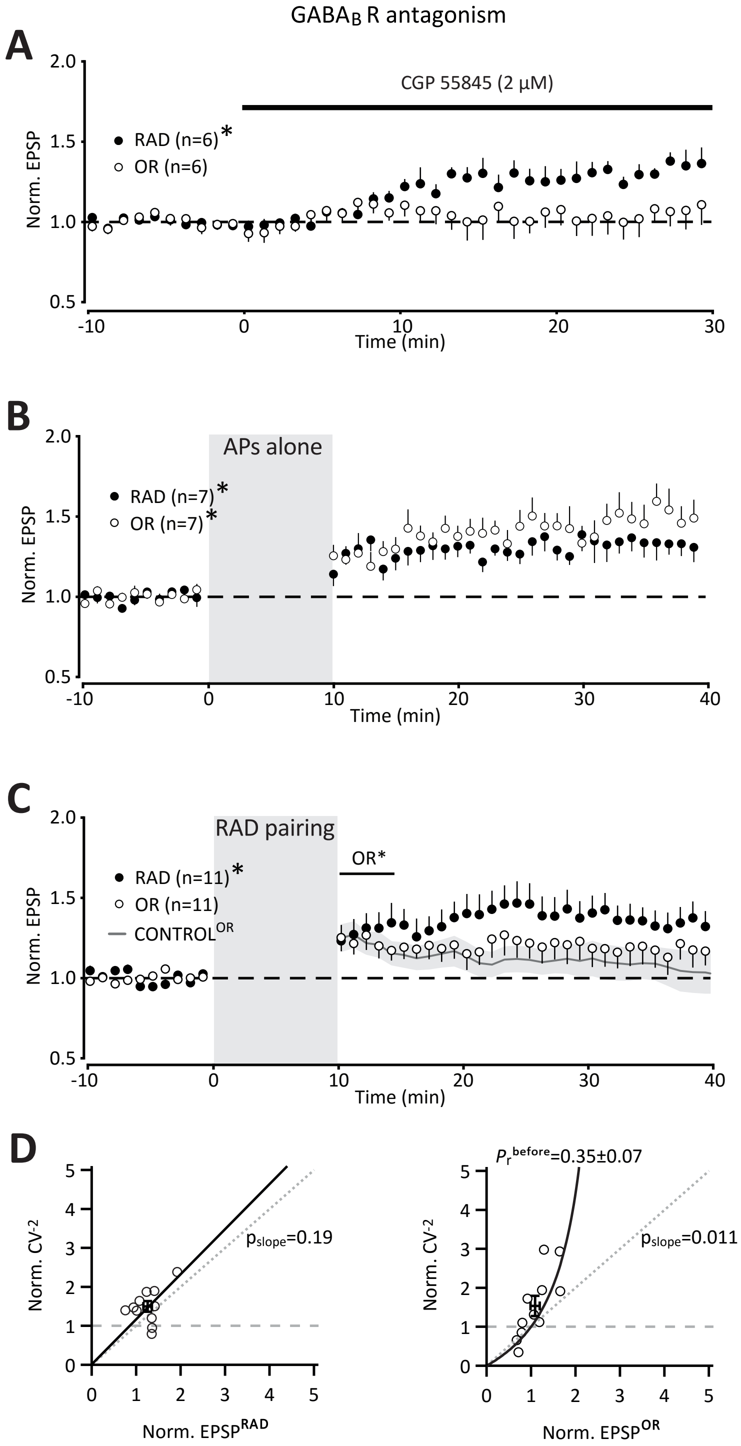

Supplementary Figure S1: $GABA_B$ receptor antagonist does not prevent input-specific potentiation. (**A**) Time course of normalized averages of EPSPs evoked either in RAD (filled) or in OR (unfilled) at 0.1 Hz before and after continuous perfusion of the $GABA_BR$ antagonist CGP 55845 (2 µM). (**B**) Time course of normalized averages of EPSPs before and after induction with APs alone in the presence of 2 µM CGP 55845. (**C**) Time course of normalized averages of EPSPs before and after RAD pairing in the presence of 2 µM CGP 55845. Control values for normalized OR EPSPs from Fig. 2A are represented by a continuous gray line ± shaded SEM. Horizontal line, labeled OR^*, indicates time interval in which amplitude change in OR was significant (*, $p < 0.05$). (**D**) Normalized CV^{-2} vs. norm. EPSPs for (C). The slope of line through the origin is different from 1 for OR (p=0.011) but not for RAD (p=0.19), indicating mainly a change in n for RAD (left) and a change in P_r for OR (right).
